# Achilles tendon structure is negatively correlated with body mass index, but not influenced by statin use: A cross-sectional study using ultrasound tissue characterization

**DOI:** 10.1371/journal.pone.0199645

**Published:** 2018-06-21

**Authors:** Agnetha de Sá, David A. Hart, Karim Khan, Alexander Scott

**Affiliations:** 1 Centre for Hip Health and Mobility, Vancouver Coastal Health Research Institute, Vancouver, Canada; 2 Department of Medicine, University of British Columbia, Vancouver, Canada; 3 McCaig Institute for Bone & Joint Health, University of Calgary, Calgary, Canada; 4 Department of Family Practice, University of British Columbia, Vancouver, Canada; 5 Department of Physical Therapy, University of British Columbia, Vancouver, Canada; Coventry University, UNITED KINGDOM

## Abstract

**Introduction:**

Statins are widely used to inhibit cholesterol production in the liver among people with hypercholesterolemia. A recent epidemiological study in the UK has shown that statin use (unlike elevated BMI) is not associated with an increased risk of Achilles tendon rupture. However, because of laboratory reports suggesting a negative influence of statins on tenocyte metabolism, we decided to directly compare the Achilles tendon structure (cross-sectional area and longitudinal collagen organization) in regular statin users compared to non-users.

**Methods:**

We conducted ultrasound tissue characterization (UTC) of the Achilles tendon in statin users and a comparison group of similar age and gender. Statin users and control participants were recruited from May 10 2015 to February 17 2017 through a cardiovascular health centre and from the general community. Cross-sectional area of the Achilles tendon and longitudinal collagen organization (% type I echoes) were assessed using quantitative ultrasound tissue characterization by a blinded observer at a predetermined location (2 cm proximal to the calcaneus).

**Results:**

Sixty-six individuals who were either taking statins for at least one year (ST, n = 33) or a comparison group who had never taken statins (CG, n = 33) were included in the study. The Achilles tendon cross-sectional area (ST 59.7 (13) mm^2^, CG 59.9 (8.5) mm^2^) and proportion of echo-type I patterns [ST 70 (10)%, CG 74 (13)%] were equivalent in the two groups. In contrast, there was a negative correlation between BMI (r_s_ = -0.25, p = 0.042) and type I echo values. Obese individuals demonstrated a significantly lower percentage of type I echoes (62 (11)%) than individuals of normal body mass index (73 (10)% p<0.05).

**Conclusion:**

These findings demonstrate that there is no evidence of a negative statin influence on Achilles tendon structure. Given earlier reports that the risk of Achilles injury is equivalent in statin users and non-users, weightbearing exercise may be prescribed without placing the Achilles tendon at a higher risk of injury than among the general population. The results of this study are consistent with the known negative effects of elevated BMI on tendon structure, suggesting that an assessment of the Achilles tendons prior to prescribing weightbearing exercise may be prudent in obese individuals.

## Introduction

Elevated LDL-C (blood cholesterol in a complex with low density lipoprotein, LDL) is a well characterized risk factor for cardiovascular disease, including serious events such as stroke or heart attack.[1] Therapies recommended to reduce LDL-C include lifestyle modification (diet, smoking cessation) and aerobic exercise which may include tendon loading activities such as walking, jogging or running.[2,3]

In addition to prescription of exercise and lifestyle modification, statins are a widely used class of medications which can inhibit cholesterol production in the liver and thereby lead to lowered LDL-C.[1] Statins are therefore commonly used in the management of individuals with hypercholesterolemia, and are generally well tolerated. However, a minority of individuals experience side effects which can include diabetes mellitus and myalgia.[4]

Several case reports (e.g. [5], and a formal review of incidents reported in a pharmacovigilance database,[6] have suggested that Achilles tendon rupture might be among the side effects of statins. In keeping with this possibility, a number of laboratory studies have demonstrated a direct impairment of collagen metabolism in response to statin exposure, including reduced type I collagen levels and increased expression and activity of several matrix metalloproteinases.[7,8,9] However, the best evidence available to date comprises a propensity-score matched study of over 500,000 statin-users in the UK: in that study, statin use was not associated with an increased risk of Achilles tendon rupture.[10] In their discussion, the authors noted that their study could not exclude the possibility that statins might lead to milder tendon injury such as tendinopathy.[10] To our knowledge, no study to date has directly examined the Achilles tendon for signs of subclinical degeneration in regular statin users.

Ultrasound tissue characterization (UTC) uses automated algorithms to analyze three-dimensional digital images of a tendon, and assigns each pixel a value from I to IV, with higher values representing more highly aligned collagen, as indicated by stability of pixel brightness across adjacent transverse scans. UTC is able to detect the subtle structural differences among Achilles tendons which occur with gender,[11] diabetes,[12] regional variations in anatomy or pathology [11,13], and in response to exercise [14,15], all of which are characterized by a relative reduction in % type I echoes. Using UTC, we set out to test the hypothesis that statin users would demonstrate impaired Achilles tendon structure, including cross-sectional area and longitudinal collagen organization (as indicated by UTC: % type I echoes). We also examined the relation of BMI and age with these two Achilles tendon structural variables, because they are both recognized risk factors for the development of Achilles tendon injury [16,17].

## Methods

### Setting

All study visits took place at the Centre for Hip Health and Mobility (CHHM) in Vancouver, British Columbia. Recruitment and data collection occurred from May 10 2015 to February 17 2017. The study protocol was reviewed and approved by the UBC Clinical Research Ethics Board (H14-01808).

### Participants

All participants provided written informed consent. Statin users were recruited primarily through a cardiac rehabiliation program offered by the Centre for Cardiovascular Health (a division of Vancouver Coastal Health). To be included, participants had to have been taking any type of statin for at least one year. We excluded participants who had taken oral corticosteroids or fluoroquinolones in the past year, had any condition which would impact Achilles tendon health including diabetes, gout, rheumatoid arthritis, whose BMI was over 35 kg/m2, who had peripheral edema in the lower extremities, who had pain or mobility issues (e.g. the use of a cane or walker), or who were not interested or committed to attending the study visit. Eligible participants in the statin group were informed about the study through word of mouth by clinic staff, or by mail and phone. Statin users in the general community were also recruited by placing posters placed around the community. Participants in the comparison group of statin non-users had to have never taken a statin, and the same exclusion criteria applied. The comparison group was recruited from the Centre for Cardiovascular Health in person and through mail/phone contact, and additional comparison group participants were recruited by word of mouth recruiting at our research centre, at Vancouver community groups (e.g. Prime Timers, West Ender), and through posters placed around the community and online.

### Variables

#### Demographic variables

We measured participants’ weight and height at the time of the ultrsaound scan, and asked them their age in years. Participants were categorized using the short form of the International Physical Acticvity Questionnaire (IPAQ) as engaging in low, medium, or high levels of physical activity. The IPAQ questionnaire asks people to report on their physical activity levels over the preceding seven days. This questionnaire demonstrates excellent reliability [18], although the limitations of using self-report measures to characterize physical activity levels, particuarly among older populations such as in the current study, are acknowledged [19]; people tend to over-report their physical activity levels.

#### Collagen organization

Subjects lay prone on an examination bed with their feet placed on a foot and ankle stabilizer to stabilize and position the Achilles tendon. Both the left and right tendons were scanned; each tendon was scanned in duplicate. A 10MHz linear array probe (Smartprobe 10L5; Terason 2000, Teratech, USA) was connected to a robotic tracker (UTC technologies); the tracker was clamped to a pole to prevent movement artefact. The tracker was programmed to capture multiple transverse 2-D images every 0.2mm over a total Achilles tendon length of 12cm. The images were collected and stored on a computer (MacbookPro) running UTC imaging algorithms to produce a 3-D image for tomographic visualization in the transverse, sagittal, and coronal planes; the algorithms analyzed pixel stability in the longitudinal plane using a window size of 25 to discriminate four echo types [19], [20]. The more longituidnally well-organized the collagen, the greater the type I echo percentage. The examiner determined the landmark (2cm from the calcaneal insertion) in the sagittal plane. The tendon border was outlined (referred to as a contour) at 2mm proximal and distal to the landmark. The two contours were interpolated to create a 4mm long region of interest used for tissue characterization. The percentage of type I echoes were calculated within the region of interest. The test-retest reliability of type I echo percentage on separate days, based on a study of 10 individuals, yielded an ICC of 0.90.

#### Achilles tendon cross-sectional area

Using the same ultrasound scans generated as described above, the borders of the Achilles tendon was contoured in the transverse plane and the cross-sectional area image analyzed in ImageJ. The distance measures provided by the UTC were calibrated in ImageJ against scans of a phantom which contained regularly spaced 0.1mm diameter metal pins (CIRS 050).

#### Attempts to limit bias

The study was designed and the report was prepared using the Strobe checklist for reporting of cohort studies [20]. We attempted to limit sampling bias by recruiting the comparison group from the same clinic as the statin users, although it was necessary to supplement this by recruiting from the community as well. To limit sex/gender and age bias, we decided *a priori* to assemble equivalent sized ST and CG groups with equal numbers of males and females and of similar age. To limit the possibility of assessor bias during ultrasound scanning, we employed a “hands-off” approach as described above, where the ultrasound probe was clamped into position and not manipulated by the researcher. To control for bias during analysis, all images were de-identified and analyzed in a batch containing scans from statin and control groups, to ensure the researcher was blind to participant group.

#### Study size

We planned to recruit during a time period which was consistent with the timeline of the first author’s MSc program timeline, and all eligible participants who were identified during that time period were enrolled. Assuming that the variability for cross-sectional area observed in this study (10.8 mm^2^) represents the true population variability, in order to detect a difference in cross-sectional area of 20% between the statin users and comparison group with a p-value of 0.01, a two-sided independent samples t-test would have 95% power with a sample of 30 per group.

#### Statistical analysis

The experimental unit in this study was the individual, who either was or was not exposed to statins. It was assumed that the ingestion of statins could affect either the right or left tendon, therefore the values of the two tendons were averaged for each structural variable. We used the Shapiro-Wilks test to determine whether data were normally distributed. For normally distributed data (age, BMI, cross-sectional area), we used parametric tests (independent t-tests, and Pearson’s correlation coefficient), and reported means and standard deviations. For non-normally distributed data (type I echoes) we used non-parametric tests (Mann-Whitney U test, Spearman’s Rho) and reported the mean and interquartile range (IQR). We used two-tailed tests (p = 0.05) to compare the tendon structural variables between statin users and the comparison group, because one could argue either that statin use would lead to tendon atrophy, or to injury and therefore expansion of the cross-sectional area. Because we expected women’s tendons to be smaller and contain a lower precentage of type I echoes than men’s tendons, we conducted 1-tailed tests to examine these comparisons, as an internal control of the measurement techniques. We also calculated correlation coefficients (Pearson’s r or Spearman’s r_s_, as stated above) to examine the potential relation of BMI and age to the dependent variables in the combined cohort (ST and CG). Upon noting a correlation between BMI and type I echoes, we conducted a Kruskal-Wallis test to compare the distribution of type I echoes among those of normal BMI (18–24.9), overweight (25.0–29.9), and obese (30–35). Finally, we examined duration of time on statins (self-reported), and found this data to be non-normally distributed; we applied Spearman’s rank-order test to see if this variable was correlated with tendon cross-sectional area or type 1 echoes. All results are reported to two significant digits.

## Results

### Participants

#### Recruitment

Through the Cardiac Rehabilitation Centre, 378 patients were approached in person (n = 250) or were mailed the study information (n = 128). Of those who were approached in person, 224 met one or more exclusion criteria, 2 were interested and signed the consent form but then withdrew before participating, and 24 participants (21 ST and 3 CG) enrolled. An additional 129 eligible participants were mailed study information and then contacted by phone: 92 were not interested, 6 could not be reached, 10 met one or more exclusion criteria, and 21 participants (13 ST users and 8 CG) enrolled. Twenty-four individuals responded to paper or online advertisements: 4 met one or more exclusion criteria, 2 statin non-users were not enrolled because they were not the desired gender to achieve balanced groups, and 18 participants (1 ST, 17 CG) enrolled. Word of mouth recruitment led us to screen an additional 18 people, of which 8 participants (controls) met the study criteria and enrolled. Prior to any analysis and without any reference to any details of their study files, 2 very elderly statin users were removed because CG participants in the same age range had not been recruited, and 3 CG participants (women) were removed to maintain gender balance in the ST and CG groups. This recruitment method resulted in the inclusion of 33 statin users and 33 in the comparison group. Data from all 66 subjects was complete (no missing data) and all was included in the analysis.

#### Demographic and descriptive data

The subject data is shown in Table 1. Data from all included participants was analyzed, with no missing data. All of the statin users (n = 33) stated that they were consistently taking their statin (adherent to treatment) and had been on their statin for at least a year. The median duration of statin use was 4.5 years (IQR 6.25), and this duration was not corelated to tendon cross-sectional area or % Type 1 echoes. All participants had normal cholesterol levels on their last available lipid test. All of the control subjects (n = 33) had never taken a statin medication.

**Table 1:** Comparison of statin users and a comparison group of statin non-users.

Variable	All participants	Statin users	Comparison group	p-value*
N	66	33	33	
Age, years: mean (SD)	66 (9)	69 (10)	63 (8)	**0.0054**
Men	58	29	29	
Women	8	4	4	
BMI, kg/m2: mean (SD)	27 (3.7)	27 (3.3)	27 (4.1)	0.61
Physical activity levels: low, moderate, high	7, 31, 28	4, 14, 15	3, 17, 13	
Tendon cross-sectional area, mm^2^: mean (SD)	60 (11)	60 (13)	60 (9)	0.95
% Type I echoes: median (IQR)	72 (13)	70 (10)	74 (13)	0.059

BMI: Body mass index. SD: Standard deviation. IQR: Interquartile range.

* For normally distributed data (age, BMI, cross-sectional area), independent t-tests were used. For non-normally distributed data (% Type I echoes), a Mann-Whitney U test was used.

As expected, women’s Achilles tendons were slightly smaller (54 (8.9) mm^2^ vs 61 (11) mm^2^, p = 0.064) and contained a lower percentage of type I echoes (57 (13) % vs 70 (9.2), p = 0.0063), despite being of equivalent age, BMI and activity level as men.

As seen in Table 1, the statin group was slightly older than the comparison group (despite our effort to recruit a control group of similar age to the statin users). However, age was not correlated with either tendon cross-sectional area (r = -0.15, n = 66) or percentage type I echoes (r_s_ = -0.16, n = 66).

**Fig 1:**
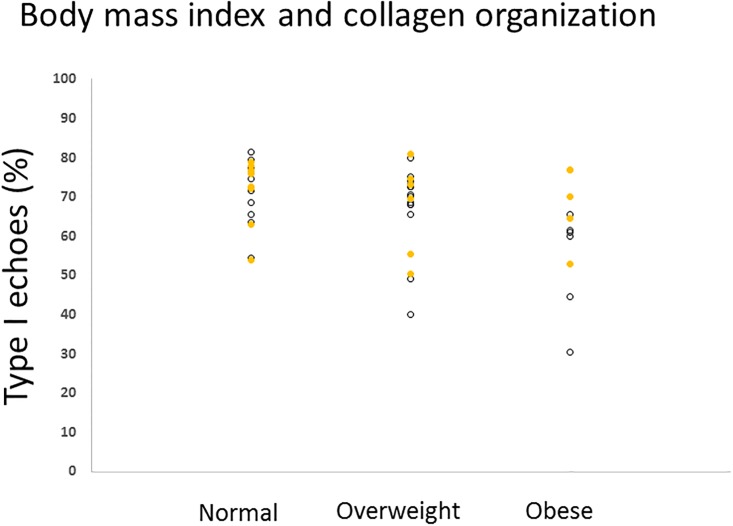
Body mass index and collagen organization. The entire cohort (n = 66) was categorized as having a body composition that was normal (18.5 to 24.9, n = 24), overweight (25 to 29.9, n = 21) or obese (30+, n = 11).[21] Statin users are shown as open circles, controls are yellow. Obese individuals (comprising 5 male statin-users, 1 female statin user, and 5 male comparison participants,) demonstrated a reduced collagen organization (Kruskal-Wallis H = 7.4, df = 2, p = 0.02) compared to those of normal body weight.

There was a negative correlation between BMI and the percentage of type I echoes (r_s_ = -0.25, p = 0.011, n = 66). Because BMI was not different between statin users and the comparison group (Table 1), we examined the influence of BMI category in the entire cohort (n = 66) according to standard criteria (Fig 1). This analysis demonstrated that the Achilles tendons from obese individuals (n = 11) displayed a reduced percentage of type I echoes.

## Discussion

This study did not identify any influence of statin use on Achilles tendon cross-sectional area or collagen organization (UTC: percentage type I echoes). These results are encouraging for health professionals working in cardiovascular rehabilitation who may wish to prescribe load-bearing exercise as part of a multidisciplinary intervention for people with hypercholesterolemia.[2,3]

Using ultrasound tissue characterization, we detected a negative moderate correlation between BMI and collagen organization in the Achilles tendon, which was an expected finding given that increasing BMI is a recognized risk factor for Achilles tendinopathy [22] and tendon rupture [17], both of which are often preceded by detectable degenerative changes in the Achilles tendon collagenous matrix.[23] The mechanism for the relation between excessive body weight and Achilles tendon pathology has not yet been elucidated, but may involve both mechanical and biological effects (reviewed in [24]).

This study is limited by its cross-sectional nature: statin-users were assessed at least 1 year after commencing lipid-lowering therapy. It is possible that participants who initially presented with dyslipidemia may have experienced an improvement in tendon structure as lipid levels normalized in response to statins, exercise and lifestyle management.[25] Furthermore, it would have been helpful to know whether tendon properties were related to lipid levels at baseline, and to examine the relation of lipid profile with tendon structure in the comparison group. Although not the primary focus of this study, such a relation would have been of interest given that LDL-C and TC levels are, on average, higher in people with tendon pathology.[26] The mechanism may involve an inhibitory effect of oxidized LDL on tenocytes.[27]

The generalizability of this study is limited to relatively sedentary individuals of advanced age. We cannot extrapolate with regard to the potential impact of statins in younger or middle-aged individuals engaged in more elite-level sporting activities who are more prone to develop Achilles tendinopathy. This group may warrant separate focus in a future study.

In conclusion, we found no difference in the Achilles tendon structure of statin users compared with a group of non-users, as visualized with ultrasound tissue characterization. These results are in keeping with a large, well-controlled epidemiological study which demonstrates a lack of negative impact of statins on tendon health.[10]

## Supporting information

S1 Table Primary data used to test the hypotheses in the current study. (XLSX)

## References

[1] Stamler J, Wentworth D, Neaton JD Is relationship between serum cholesterol and risk of premature death from coronary heart disease continuous and graded? Findings in 356,222 primary screenees of the Multiple Risk Factor Intervention Trial (MRFIT). JAMA 1986 256: 2823–2828. 3773199

[2] Stone NJ, Robinson JG, Lichtenstein AH, Bairey Merz CN, Blum CB, Eckel RH, et al 2013 ACC/AHA guideline on the treatment of blood cholesterol to reduce atherosclerotic cardiovascular risk in adults: a report of the American College of Cardiology/American Heart Association Task Force on Practice Guidelines. J Am Coll Cardiol 2014 63: 2889–2934. doi: 10.1016/j.jacc.2013.11.002 24239923 10.1016/j.jacc.2013.11.002

[3] Wasfy MM, Baggish AL Exercise Dose in Clinical Practice. Circulation 2016 133: 2297–2313. doi: 10.1161/CIRCULATIONAHA.116.018093 27267537 10.1161/CIRCULATIONAHA.116.018093 PMC4902280

[4] Thompson PD, Panza G, Zaleski A, Taylor B Statin-Associated Side Effects. J Am Coll Cardiol 2016 67: 2395–2410. doi: 10.1016/j.jacc.2016.02.071 27199064 10.1016/j.jacc.2016.02.071

[5] Chazerain P, Hayem G, Hamza S, Best C, Ziza JM Four cases of tendinopathy in patients on statin therapy. Joint Bone Spine 2001 68: 430–433. 11707010 10.1016/s1297-319x(01)00300-1

[6] Marie I, Delafenetre H, Massy N, Thuillez C, Noblet C Tendinous disorders attributed to statins: a study on ninety-six spontaneous reports in the period 1990–2005 and review of the literature. Arthritis Rheum 2008 59: 367–372. doi: 10.1002/art.23309 18311771 10.1002/art.23309

[7] de Oliveira LP, Vieira CP, Da Re Guerra F, de Almeida Mdos S, Pimentel ER Statins induce biochemical changes in the Achilles tendon after chronic treatment. Toxicology 2013 311: 162–168. doi: 10.1016/j.tox.2013.06.010 23831763 10.1016/j.tox.2013.06.010

[8] Eliasson P, Svensson RB, Giannopoulos A, Eismark C, Kjaer M, Schjerling P, et al Simvastatin and atorvastatin reduce the mechanical properties of tendon constructs in vitro and introduce catabolic changes in the gene expression pattern. PLoS One 2017 12: e0172797 doi: 10.1371/journal.pone.0172797 28264197 10.1371/journal.pone.0172797 PMC5339395

[9] Kuzma-Kuzniarska M, Cornell HR, Moneke MC, Carr AJ, Hulley PA Lovastatin-Mediated Changes in Human Tendon Cells. J Cell Physiol 2015 230: 2543–2551. doi: 10.1002/jcp.25010 25846724 10.1002/jcp.25010 PMC4832302

[10] Spoendlin J, Layton JB, Mundkur M, Meier C, Jick SS, Meier CR The Risk of Achilles or Biceps Tendon Rupture in New Statin Users: A Propensity Score-Matched Sequential Cohort Study. Drug Saf 2016 39: 1229–1237. doi: 10.1007/s40264-016-0462-5 27677637 10.1007/s40264-016-0462-5

[11] Wezenbeek E, Mahieu N, Willems TM, Van Tiggelen D, De Muynck M, De Clercq D, et al What does normal tendon structure look like? New insights into tissue characterization in the Achilles tendon. Scand J Med Sci Sports 2017 27: 746–753. doi: 10.1111/sms.12706 27367438 10.1111/sms.12706

[12] de Jonge S, Rozenberg R, Vieyra B, Stam HJ, Aanstoot HJ, Weinans H, et al Achilles tendons in people with type 2 diabetes show mildly compromised structure: an ultrasound tissue characterisation study. Br J Sports Med 2015 49: 995–999. doi: 10.1136/bjsports-2014-093696 25586910 10.1136/bjsports-2014-093696

[13] Wynter Bee W, Ubhi J, Kumar B Subcategories of tendinopathy using ultrasound tissue characterization (UTC): dorsal mid-portion achilles tendinopathy is more severe than ventral achilles tendinopathy. Br J Sports Med (in press). 10.1136/bjsports-2017-097827.4 28490458

[14] Rosengarten SD, Cook JL, Bryant AL, Cordy JT, Daffy J, Docking SI Australian football players' Achilles tendons respond to game loads within 2 days: an ultrasound tissue characterisation (UTC) study. Br J Sports Med 2015 49: 183–187. doi: 10.1136/bjsports-2013-092713 24735840 10.1136/bjsports-2013-092713

[15] Waugh CM, Alktebi T, de Sa A, Scott A Impact of rest duration on Achilles tendon structure and function following isometric training. Scand J Med Sci Sports 2018 28: 436–445. doi: 10.1111/sms.12930 28603874 10.1111/sms.12930

[16] Ganestam A, Kallemose T, Troelsen A, Barfod KW Increasing incidence of acute Achilles tendon rupture and a noticeable decline in surgical treatment from 1994 to 2013. A nationwide registry study of 33,160 patients. Knee Surg Sports Traumatol Arthrosc 2016 24: 3730–3737. doi: 10.1007/s00167-015-3544-5 25697284 10.1007/s00167-015-3544-5

[17] Claessen FM, de Vos RJ, Reijman M, Meuffels DE Predictors of primary Achilles tendon ruptures. Sports Med 2014 44: 1241–1259. doi: 10.1007/s40279-014-0200-z 24929701 10.1007/s40279-014-0200-z

[18] Silsbury Z, Goldsmith R, Rushton A Systematic review of the measurement properties of self-report physical activity questionnaires in healthy adult populations. BMJ Open 2015 5: e008430 doi: 10.1136/bmjopen-2015-008430 26373402 10.1136/bmjopen-2015-008430 PMC4577932

[19] Forsen L, Loland NW, Vuillemin A, Chinapaw MJ, van Poppel MN, Mokkink LB, et al Self-administered physical activity questionnaires for the elderly: a systematic review of measurement properties. Sports Med 2010 40: 601–623. doi: 10.2165/11531350-000000000-00000 20545382 10.2165/11531350-000000000-00000

[20] von Elm E, Altman DG, Egger M, Pocock SJ, Gotzsche PC, Vandenbroucke JP The Strengthening the Reporting of Observational Studies in Epidemiology (STROBE) Statement: guidelines for reporting observational studies. Int J Surg 2014 12: 1495–1499. doi: 10.1016/j.ijsu.2014.07.013 25046131 10.1016/j.ijsu.2014.07.013

[21] Physical status: the use and interpretation of anthropometry. Report of a WHO expert committee. 1995. 8594834

[22] Scott RT, Hyer CF, Granata A The correlation of Achilles tendinopathy and body mass index. Foot Ankle Spec 2013 6: 283–285. doi: 10.1177/1938640013490019 23687344 10.1177/1938640013490019

[23] McAuliffe S, McCreesh K, Culloty F, Purtill H, O'Sullivan K Can ultrasound imaging predict the development of Achilles and patellar tendinopathy? A systematic review and meta-analysis. Br J Sports Med 2016 50: 1516–1523. doi: 10.1136/bjsports-2016-096288 27633025 10.1136/bjsports-2016-096288

[24] Scott A, Zwerver J, Grewal N, de Sa A, Alktebi T, Granville DJ, et al Lipids, adiposity and tendinopathy: is there a mechanistic link? Critical review. Br J Sports Med 2015 49: 984–988. doi: 10.1136/bjsports-2014-093989 25488953 10.1136/bjsports-2014-093989 PMC4518755

[25] Kolovou G, Daskalova D, Mastorakou I, Anagnostopoulou K, Cokkinos DV Regression of Achilles tendon xanthomas evaluated by CT scan after hypolipidemic treatment with simvastatin. A case report. Angiology 2004 55: 335–339. doi: 10.1177/000331970405500314 15156269 10.1177/000331970405500314

[26] Tilley BJ, Cook JL, Docking SI, Gaida JE Is higher serum cholesterol associated with altered tendon structure or tendon pain? A systematic review. Br J Sports Med 2015 49: 1504–1509. doi: 10.1136/bjsports-2015-095100 26474596 10.1136/bjsports-2015-095100 PMC4680137

[27] Grewal N, Thornton G, Behzad H, Sharma A, Lu A, Zhang P, et al Accumulation of Oxidized LDL in the Tendon Tissues of C57BL/6 or Apolipoprotein E Knock-Out Mice That Consume a High Fat Diet: Potential Impact on Tendon Health. PLoS One 2014 9: e114214 doi: 10.1371/journal.pone.0114214 25502628 10.1371/journal.pone.0114214 PMC4264764

